# Supplementary material for: Association between Visceral Adiposity Index, Binge Eating Behavior, and Grey Matter Density in Caudal Anterior Cingulate Cortex in Severe Obesity
Source: Brain Sci. 2021 Aug 31;11(9):1158. doi: 10.3390/brainsci11091158 (PMC8468041; doi:10.3390/brainsci11091158)
Supplement: Supplementary file 1 [file brainsci-11-01158-s001.zip › brainsci-1340481-supplementary.pdf]

Association between visceral adiposity index, binge eating behavior and gray matter volume in caudal anterior cingulate cortex in severe obesity

Sylvain Iceta[1,2,*], Mahsa Dadar[3], Justine Daoust[1,2], Anais Scovronec[1,2], Vicky Leblanc[2], Melissa Pelletier[1], Laurent Biertho[4], Andre Tchernof[1,2], Catherine Begin[1,5,6], Andreanne Michaud[1,2,5*]

[1]Research Center of the Quebec Heart and Lung Institute, Université Laval, Québec, Canada
[2]School of Nutrition, Université Laval, Québec, Canada
[3]CERVO Brain Research Center, Centre intégré universitaire santé et services sociaux de la Capitale Nationale, Université Laval, Québec, Canada
[4]Département de chirurgie générale, Institut universitaire de cardiologie et de pneumologie de Québec, Université Laval, Québec, Canada
[5]Centre de Recherche Nutrition, Santé et Société (NUTRISS), Institut sur la Nutrition et les Aliments Fonctionnels (INAF), Université Laval, Québec, Canada
[6]School of Psychology, Laval University, Quebec City, Quebec, Canada
*Corresponding authors at:
Andréanne Michaud, Ph.D., andreanne.michaud@criucpq.ulaval.ca
and Sylvain Iceta, M.D., Ph.D., sylvain.iceta.1@ulaval.ca

Supplemental Material :

Table S1: Clinical characteristics, anthropometric and biological parameters of participants.

Table S2: Comparison of grey matter density in selected ROIs in men and wom-en with high- versus low- visceral adiposity index (VAI).

Table S3: Comparison of grey matter density in selected ROIs between participants with high- versus low-visceral adiposity index (VAI) using a generalized linear model with age as covariate.

Supplemental material Table S1: Clinical characteristics, anthropometric and biological parameters of participants.

	OVERALL			WOMEN			MEN			
	n = 79			*n* = 60			*n* = 19			
				Mean or *n*		SD or %	Mean or *n*		SD or %	*p* value
Age (yr)	44.5	±	8.7	43.4	±	8.8	47.8	±	7.7	0.056
Type 2 Diabetes (%)	18	-	22.8	13	-	21.7	5	-	26.3	0.674*
**Anthropometrics**										
BMI (kg/m²)	43.6	±	4.0	44.1	±	3.8	42.1	±	4.1	0.052
Waist circumference (cm)	129	±	9	129	±	9	132	±	10	0.191
Hips circumference (cm)	132	±	10	134	±	9	126	±	11	0.002
Neck circumference (cm)	41	±	4	40	±	2	46	±	2	< 0.001
Waist-to-hip ratio	1.0	±	0.1	1.0	±	0.1	1.1	±	0.1	< 0.001
Waist-to-height ratio	0.8	±	0.1	0.8	±	0.1	0.7	±	0.1	0.005
Percentage of fat mass (%)	48.8	±	5.7	51.4	±	2.4	40.7	±	5.7	< 0.001
Body fat mass index (kg/m²)	21.4	±	3.8	22.7	±	2.7	17.3	±	3.9	< 0.001
Visceral adiposity index	2.43	±	1.16	2.48	±	1.11	2.25	±	1.34	0.455
**Biological parameters**										
Triglycerides (mmol/L)	1.50	±	0.62	1.45	±	0.50	1.67	±	0.89	0.327
Total cholesterol (mmol/L)	4.45	±	0.91	4.51	±	0.84	4.27	±	1.12	0.312
HDL cholesterol (mmol/L)	1.22	±	0.29	1.26	±	0.30	1.10	±	0.22	0.036
LDL cholesterol (mmol/L)	2.54	±	0.80	2.59	±	0.74	2.40	±	0.96	0.386
Apolipoprotein B (g/L)	0.94	±	0.22	0.94	±	0.20	0.92	±	0.27	0.743
Fasting glucose (mmol/L)	6.2	±	1.6	6.3	±	1.8	6.1	±	0.9	0.792
Insulin (pmol/L)	169.2	±	96.1	160.4	±	97.5	196.8	±	88.2	0.151
HOMA-IR index	8.1	±	5.5	7.8	±	5.8	9.0	±	4.5	0.395
HbA1c (%)	5.7	±	0.9	5.8	±	1.0	5.6	±	0.8	0.471
TSH (mU/L)	2.61	±	1.32	2.63	±	1.30	2.53	±	1.41	0.790

*SD : Standard Deviation; BMI : body mass index; HDL : high-density lipoprotein ; LDL : low-density lipoprotein; HOMA-IR : homeostatic model assessment for insulin resistance; HbA1c : hemoglobin A1c or glycated hemoglobin ; TSH : thyroid stimulating hormone; * Fisher's Exact Test.*

Supplemental material Table S2: Comparison of grey matter density in selected ROIs between participants with high- versus low-visceral adiposity index (VAI).

	MEN								WOMEN							
	LOW-VAI			HIGH-VAI					LOW-VAI			HIGH-VAI				
	n = 10			*n* = 9					*n* =32			*n* = 28				
	Mean	±	SD	Mean	±	SD	Bonferroni-adjusted *p*-value*	Unadjusted *p*-value*	Mean	±	SD	Mean	±	SD	Bonferroni-adjusted *p*-value	Unadjusted *p*-value
Insula	0.657	±	0.053	0.698	±	0.039	*0.318*	0.053	0.707	±	0.042	0.696	±	0.066	*2.634*	0.439
Orbitofrontal cortex	0.504	±	0.042	0.504	±	0.039	*3.294*	0.549	0.534	±	0.033	0.525	±	0.049	*2.538*	0.423
Caudal anterior cingulate cortex	0.577	±	0.089	0.584	±	0.064	*5.994*	0.999	0.616	±	0.077	0.559	±	0.082	*0.042*	0.007
Rostral anterior cingulate cortex	0.600	±	0.102	0.614	±	0.081	*3.624*	0.604	0.603	±	0.102	0.613	±	0.086	*4.176*	0.696
Dorsolateral prefrontal cortex	0.505	±	0.053	0.517	±	0.054	*3.966*	0.661	0.536	±	0.047	0.513	±	0.046	*0.312*	0.052
Ventromedial prefrontal cortex	0.489	±	0.074	0.457	±	0.066	*3.294*	0.549	0.508	±	0.052	0.490	±	0.068	*1.590*	0.265

*VAI mean value for men: 2.25 and for women: 2.48; SD: Standard Deviation; * Independent-Samples Mann-Whitney U Test.*

Supplemental material Table S3: Comparison of grey matter density in selected ROIs between participants with high- versus low-visceral adiposity index (VAI) using a generalized linear model with age as covariate

	MEN								WOMEN							
	LOW-VAI			HIGH-VAI					LOW-VAI			HIGH-VAI				
	n = 10			*n* = 9					*n* =32			*n* = 28				
	Mean	±	SD	Mean	±	SD	Bonferroni-adjusted *p*-value*	Unadjusted *p*-value*	Mean	±	SD	Mean	±	SD	Bonferroni-adjusted *p*-value	Unadjusted *p*-value
Insula	0.660	±	0.016	0.695	±	0.017	*0.996*	0.166	0.708	±	0.009	0.694	±	0.010	*1.704*	0.284
Orbitofrontal cortex	0.513	±	0.012	0.494	±	0.013	*1.932*	0.322	0.534	±	0.007	0.525	±	0.008	*2.304*	0.384
Caudal anterior cingulate cortex	0.589	±	0.025	0.570	±	0.027	*3.822*	0.637	0.617	±	0.013	0.557	±	0.014	*0.018*	0.003
Rostral anterior cingulate cortex	0.607	±	0.031	0.606	±	0.033	*5.886*	0.981	0.604	±	0.017	0.612	±	0.018	*4.548*	0.758
Dorsolateral prefrontal cortex	0.512	±	0.017	0.509	±	0.018	*5.412*	0.902	0.537	±	0.008	0.512	±	0.009	*0.198*	0.033
Ventromedial prefrontal cortex	0.502	±	0.022	0.443	±	0.023	*0.576*	0.096	0.508	±	0.011	0.490	±	0.011	*1.434*	0.239

*VAI mean value for men: 2.25 and for women: 2.48; SD: Standard Deviation; mean: estimated marginal means; ** generalized linear model with age as covariate.
